# A predictive quality assurance model for patient‐specific gamma passing rate of hyperarc‐based stereotactic radiotherapy and radiosurgery of brain metastases

**DOI:** 10.1002/acm2.70225

**Published:** 2025-08-21

**Authors:** Shane McCarthy, Brent Harrison, Damodar Pokhrel

**Affiliations:** ^1^ Medical Physics Graduate Program, Department of Radiation Medicine University of Kentucky Lexington Kentucky USA; ^2^ College of Engineering University of Kentucky Lexington Kentucky USA

**Keywords:** gamma passing rate, hyperarc, measurement‐based quality assurance, patient‐specific quality assurance, predictive machine learning, stereotactic radiosurgery/radiotherapy

## Abstract

**Objective:**

Measurement‐based patient specific quality assurance (PSQA) is an increasingly debated topic among medical physicists. Developments like online adaptive radiotherapy and same‐day stereotactic treatments limit the time to do measurement‐based PSQA. Herein, we develop a predictive machine learning model to supplement PSQA by predicting the gamma passing rate (GPR) per stereotactic arc. This streamlines PSQA, providing planners the insight to replan potentially sub‐optimal plans, to mitigate machine time inefficiencies.

**Methods:**

122 patients that had previously received HyperArc stereotactic radiosurgery/radiotherapy on a TrueBeam LINAC (Millenium 120 MLCs, 6MV‐FFF) were used to generate a long short‐term memory (LSTM) recurrent neural network to predict the GPR for a 2%/2 mm criteria. GPRs were discretized into three classes: Ideal (≥95%), Investigate [85%–95%), and Replan (<85%). In total, 468 VMAT arcs were used for this model with a class distribution of 370 (Ideal), 65 (Investigate), and 33 (Replan). To counteract the imbalanced data, the minority classes were over‐sampled using synthetic minority over‐sampling technique to generate a balanced dataset. The LSTM model was trained in Python with an 80‐20 training‐testing stratified split. Individual class sensitivity and specificity were recorded following a one versus all method. The final model was deployed clinically through Eclipse Scripting.

**Results:**

The model demonstrated the following (sensitivity, specificity) for the testing data: Ideal (78.4%, 87.2%), Investigate (75.7%, 89.9%), and Replan (93.2%, 96.6%). The primary focus of this model is to identify failing beams and allow the planner to address this prior to running the PSQA, as such the Replan class was the most important for evaluation. A sensitivity of 93.2% indicates that the model will identify 93.2% of HyperArc plans that need to be replanned with a very high certainty due to the 96.6% specificity.

**Conclusions:**

The predictive GPR model developed within this research enables HyperArc planners to immediately assess the GPR for each stereotactic arc and preemptively replan potentially failing arcs to optimize the PSQA machine time.

## 1 INTRODUCTION

In order to maintain fast, safe and effective treatment deliveries, a time‐consuming portion of a medical physicist's job is performing measurement‐based patient‐specific quality assurance (PSQA) for all intensity modulated radiation therapy (IMRT) and volumetric modulated arc therapy (VMAT) treatment plans. These heavily modulated plans often result in exceptionally complex machine delivery sequences requiring precise harmony of several moving parts, including variable dose‐rate as a function of gantry and multi‐leaf collimator (MLC). In order to assess the ability of the machine to deliver these complex radiation treatment plans, measurement‐based PSQA has become a clinical standard for these types of treatments.^1^

Various techniques exist for performing measurement‐based PSQA through the use of film^2^, ^3^ diode arrays^4^ and portal dosimetry (PD) with an electronic portal imaging devices (EPID).^5^ At our institution, we use EPID‐based PD to perform measurement‐based PSQA with our HyperArc stereotactic radiosurgery (SRS) and radiotherapy (SRT) plans for the treatment of single‐isocenter single‐ and multi‐lesion brain metastases. Although uncommon, there are occurrences where a HyperArc plan, with its extensive use of small field segments with high dose gradients, might receive a failing gamma passing rate (GPR) and could potentially require re‐optimization or entire replanning. Our clinical action limit requires a GPR ≥ 90% at a 2%/2 mm gamma criteria and a 10% threshold to pass. This limit is based on the recommendation of Task Group 218 and the encouragement towards tighter tolerances for SRS cases.^1^ This occurrence is detrimental to clinical efficiency, requiring additional treatment planning time and excessive machine time to repeatedly perform the PSQA measurement. As the field of radiation oncology continues to progress towards shorter and shorter simulation to treatment time with several same‐day treatment methodologies being developed and the relatively recent explosion of online adaptive radiotherapy, the time in which to conduct measurement‐based PSQA is getting increasingly shorter and in the case of online adaptive radiotherapy, might not be performed at all.^6^, ^7^

Several studies have explored the possibility of supplementing this process through the inclusion of various machine learning models and algorithms to further assess the quality of the treatment plan and possibly predict the GPR.^8^, ^9^, ^10^, ^11^, ^12^, ^13^, ^14^, ^15^ This research aimed to explore a similar avenue of developing a machine learning model to supplement the PSQA process through the prediction of the GPR per stereotactic VMAT arc for HyperArc brain SRS/SRT plans. Furthermore, this research sought to fully integrate the machine learning techniques within the clinical treatment planning system (TPS) through integration within the Eclipse ecosystem to allow physicists and planners seamless access to predictive models for assessing the quality of their HyperArc plans, potentially improving the general clinical workflow through a reduction of repetitive measurements.

## 2 MATERIALS AND METHODS

### 2.1 Patients data collection

After obtaining institutional review board approval from our cancer center, a total of one hundred and twenty‐two (122) single‐isocenter single‐ and multi‐lesion brain metastases patients were gathered for this research. All patients were treated using Varian's HyperArc module on a TrueBeam LINAC (6MV‐FFF with a maximum dose rate of 1400 MU/min) equipped with the Millenium 120 (MLCs) with the 0.5 cm width leaves (at isocenter) in the center 20 cm of the treatment field. Across all SRS/SRT plans there were 332 total lesions with an average of 2.7 ± 3.0 lesions per patient ranging from 1 lesion to 20 lesions. Lesions had an average PTV size of 6.98 ± 13.76 cm^3^ ranging from 0.0 to 99.43 cm^3^. The stereotactic plans were either single‐fraction (11), three‐fraction (53), or five‐fraction (58) with prescription doses ranging from 18–24, 24–27, and 25–35 Gy, respectively.

For each plan the gamma passing rate (GPR) was extracted for each arc using Varian's Portal Dosimetry Scripting API. In total, there were four hundred and sixty‐eight (468) arcs across all plans. The stereotactic VMAT arcs were evaluated on a clinical gamma criterion of 2%/2 mm with a 10% global threshold. The average GPR was 96.1% ± 7.9% ranging from 26.7% to 100.0%.

For each arc, beam information was extracted from all control points using Eclipse Scripting API (ESAPI). For each control point, the index, meter set weight, jaw positions, gantry angle, and leaf positions were flattened into a single vector of length 127. The number of control points per arc varied between arcs, ranging from 90 to 180 control points. After data extraction, all arcs were padded with zeros to ensure a consistent tensor size for the model.

Commonly in a clinical setting, the GPR is assessed as either passing or failing. When a GPR fails, the distributions are investigated further, and an assessment of the plan is made by a qualified physicist. This process leads to an intermediate region where although a GPR might fail the established criteria, the plan could still be deemed acceptable and delivered. To account for this intermediate region, and better reflect the clinical workflow, the prediction of the GPRs was turned into a three‐class classification problem with the classes being defined as

$$Class=\left\{\begin{array}{c} Replan,\;GPR<85\%\\ Investigate,\;85\%\leq GPR<95\%\\ Ideal,\;GPR\geq 95\%. \end{array}\right.$$

The limits for each class were established experimentally during preliminary explorations to determine what worked best for the given dataset.

#### 2.1.1 Resampling

The distribution of the GPRs demonstrated a heavy left skew with the Replan class having 33 samples, the Investigate class with 65 samples, and the Ideal class with 370 samples. To help mitigate this class imbalance, synthetic minority over‐sampling technique (SMOTE)^16^ was used to over‐sample the minority classes. SMOTE was implemented through the imbalanced‐learn (v0.13.0)^17^ library within Python. After resampling each class had 370 samples for a total of 1110 samples to train and test the model with.

#### 2.1.2 Training and testing data splits

The 1110 samples were split into training and testing sets with an 80% (888 samples) to 20% (222 samples) split, respectively. A stratified split was used to ensure an equal representation of each class in both datasets.

### 2.2 The GPR model creation

#### 2.2.1 Architecture

Due to the complex sequential nature of the data, a recurrent neural network (RNN) was developed using pytorch (v2.0.1).^18^ A long short‐term memory (LSTM) network was used over a conventional RNN to help mitigate the inherent vanishing gradient problem of RNNs. After the LSTM the final output layer was fed to a linear transformation to compress the output down to three classes. The final model had a hidden size of 64 with 3 layers and no dropout probability.

#### 2.2.2 Training and testing the GPR model

The predictive GPR model was trained for 1000 epochs using 148‐fold cross validation with a batch size of 64. The validation data split was stratified with each class having 2 samples per fold. A large number of folds were used to maximize the amount of data being used to train the model during cross validation. This provided a performance estimate that was more representative of the final model trained on the entire training data, albeit with an increased variance within each validation fold. A patience value of 500 epochs was used to implement early stopping based on the validation loss value. The Adam optimizer was used with a starting learning rate of 1 × 10^−2^. Loss was evaluated with cross entropy loss. Throughout training, the sensitivity and specificity were calculated for each class following a one against all approach.

**FIGURE 1:**
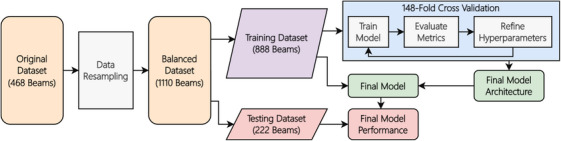
Process workflow outlining data process through model training and testing. Data was resampled using SMOTE over‐sampling of the minority classes. Training and testing data was an 80%–20% split. 148‐fold cross‐validation was used for hyperparameter fine tuning. The final model was trained on all the training data with no hold out.

The development of this predictive GPR model was intended to provide treatment planners a tool to quickly identify beams that have a high probability of resulting in a failing GPR. This would allow treatment planners to preemptively investigate or replan those beams prior to PSQA to reduce a repetitive workflow. With this in mind, the stopping point for the model training was to achieve an average validation sensitivity of at least 50% and a specificity of at least 70% for all classes. Additionally, the goal of the total accuracy for the model was to achieve an accuracy above 70%. After the results of the cross‐validation training were found to be satisfactory, the model was trained on the entire training dataset as shown in Figure 1. The final number of epochs was the average of the triggered early stopping epochs from the cross‐validation folds.

The testing data was then used to evaluate the performance of the final model. Similarly, to the validation evaluation, the model's performance on the testing data was evaluated for the sensitivity and specificity of each class following a one versus all approach.

### 2.3 TPS integration

The predictive GPR model was then converted to an Open Neural Network eXchange (ONNX) format using Python's ONNX library (v1.17.0)^19^ and then deployed in the C# language through Microsoft's ONNX Runtime (v1.8.0)^20^ to be used in conjunction with ESAPI. The model was integrated into our institution's in‐house auto‐planning software for HyperArc brain SRS/SRT treatments to be ran automatically after dose optimization as well as being developed to be launched independently if desired.

## 3 RESULTS

### 3.1 Model creation

#### 3.1.1 Model training

**FIGURE 2:**
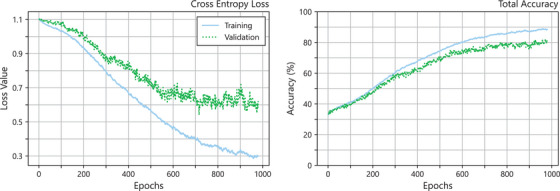
Accuracy and loss curves from the 148‐fold cross‐validation training of the predictive GPR model for HyperArc brain SRS/SRT. Validation values were averaged across all 148 folds.

The training for the predictive GPR model was an iterative process with several changes and adaptations to the training methods and techniques. The final acceptable model, as described previously, took approximately 10 h to train with 148 cross validation folds. Initially allowed to train for up to 1000 epochs, the cross‐validation folds triggered early stopping for an average of 980 epochs. Loss and accuracy curves are shown in Figure 2 for the training and averaged validation datasets across all epochs. As shown in the figure, the training loss curve drops slowly within the first 200 epochs then decreases slightly quicker for the remaining epochs, slowing down again near the cutoff epoch. The validation loss curve follows a similar trajectory, remaining close to the training loss until around the 500^th^ epoch where it begins decreasing now as rapidly as the training loss. The accuracy curves increase steadily within the first 600 epochs and slow down after that. The validation total accuracy curve passes the 70% threshold around the 500^th^ epoch and increases slowly after that to 80%.

**FIGURE 3:**
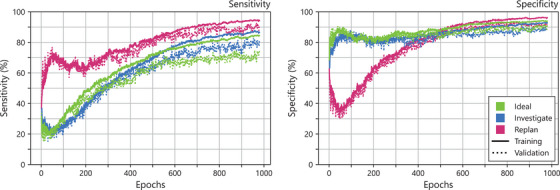
Sensitivity and specificity values for training and validation of each class during the 148‐fold cross‐validation training of the predictive GPR model for HyperArc brain SRS/SRT. Metrics were calculated following a one versus all approach. Validation values were averaged across all 148 folds.

In addition to loss and accuracy curves, the sensitivity and specificity for each class was recorded throughout each epoch following a one vs all approach as shown in Figure 3. Of particular interest was the Replan class, as shown in the figure the sensitivity for the replan class spiked slightly at the very beginning of the training then slowly increased for the remaining epochs on both the training and validation sets. Conversely, the specificity for the Replan class dips within the first 200 epochs then increases rapidly to around 90% by 500^th^ epoch. The Ideal and Investigate sensitivity curves increased gradually during the training with the validation curves slowly branching out as the training progressed. Additionally, their specificity curves quickly reached 80% within the first 100 epochs and increased slowly for the remaining epochs.

#### 3.1.2 Model testing

A final model was then trained on the entirety of the training dataset using the aforementioned cutoff of 980 epochs established from the cross‐validation folds. Model training took approximately 5 min. The final model was then evaluated against the testing dataset of 222 samples with the resulting confusion matrix reported in Table 1 along with sensitivity and specificity values.

**TABLE 1:** Confusion matrix and diagnostic metrics from the final model's performance on a testing dataset of 222 samples.

		Predicted label		
		Ideal (GPR ≥ 95%)	Investigate (85% ≤ GPR < 95%)	Replan (GPR < 85%)
Truth label	Ideal	58	12	4
	Investigate	17	56	1
	Replan	2	3	69
Sensitivity (%)		78.38	75.68	93.24
Specificity (%)		87.16	89.86	96.62

Abbreviations: GPR, gamma passing rate.

Within the testing dataset, the Replan class performed comparably to that of validation folds, achieving a sensitivity of 93.2% and a specificity of 96.6%. The Investigate class achieved a sensitivity of 75.7% and a specificity of 89.9%. The Ideal class achieved a sensitivity of 78.4% and a specificity of 87.2%. In total, the model achieved an overall accuracy of 82.4%.

**FIGURE 4:**
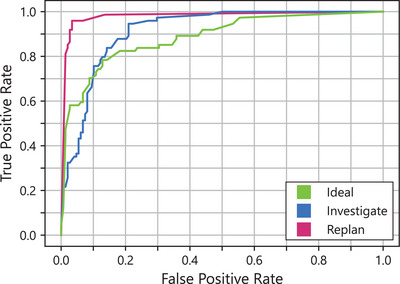
ROC curve for the multiclass predictive GPR model generated following a one versus all approach. Ideal, Investigate, and Replan AUROC scores were 0.89, 0.91, and 0.98, respectively.

A receiver operating characteristic (ROC) curve is shown in Figure 4 for the three‐class classification problem following a one‐vs‐all approach. The ROC curve is not used to determine an optimal threshold, as is common for a binary classification, but rather to provide an assessment of the model's performance for each class. Replan, Investigate, and Ideal classes’ Area Under the ROC curve (AUROC) scores were found to be 0.98, 0.91, and 0.89, respectively.

### 3.2 Eclipse integration for hyperarc SRS/SRT

**FIGURE 5:**
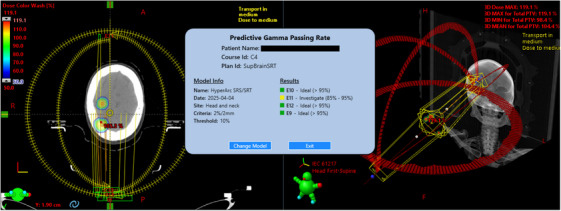
Graphical user interface of the predictive gamma passing rate application running within Eclipse's external beam planning module through ESAPI. A three‐lesion HyperArc brain SRT plan is shown.

The predictive GPR model was successfully converted to an ONNX model and integrated within the ESAPI ecosystem. The process of ensuring version compatibility across the various third‐party libraries and the constraints of Eclipse was nontrivial. A Windows Presentation Foundation application was developed for interfacing with the model clinically for HyperArc brain SRS/SRT plans. The application was designed to accept a variety of models with the intent of developing additional models for other treatment sites. The user interface, shown in Figure 5, displays the relevant patient information, model information, and the results for each beam. The application takes less than 10 s to run with the majority of the time being ESAPI loading time, the machine learning model embedded within the software is near instantaneous.

Since the creation and deployment of the application and model, 12 new HyperArc SRS/SRT cases have been created within our clinic. Across these 12 plans, there were a total 48 beams. A confusion matrix for this preliminary clinical data is shown in Table 2. Due to the small number of samples within the minority classes, nine for Investigate and only one for Replan, no statistical measures are conducted.

**TABLE 2:** Confusion matrix from the final model's performance on a clinical dataset from 12 cases with a total of 48 beams.

		Predicted label		
		Ideal (GPR ≥ 95%)	Investigate (85% ≤ GPR < 95%)	Replan (GPR < 85%)
Truth label	Ideal	32	5	1
	Investigate	4	2	3
	Replan	1	0	0

Abbreviations: GPR, gamma passing rate.

## 4 DISCUSSION

The overall performance of the predictive GPR model developed in this research was found to be acceptable for the given problem and intended application. The training goals of achieving a greater than 50% sensitivity and 70% specificity for all classes was achieved in both the cross‐validation results and the final testing results, with the final testing total accuracy achieving a value of 82.4%. The Replan class having a sensitivity of 93.2% and a specificity of 96.6% assures that when the model is being used clinically, if it identifies a beam as Replan, the planner can be assured with a high degree of confidence that the beam will fail and can justify spending the time to replan. Furthermore, the Ideal class having a specificity of 87.2% indicates the model will not be likely to produce false positives for passing beams/arcs. The Investigate class performed comparably to that of the Ideal class, with both classes performing worse than the Replan class. This is thought to be indicative of a greater separation of feature characteristics from the Replan class towards the other two classes. This observation could be used to justify declaring the Replan data as possible outliers and introducing a lower limit to the GPRs considered for model training, potentially improving the model's ability to discriminate among the Ideal and Investigate classes more accurately.

This model is not intended to replace measurement based PSQA, as such, all brain SRS/SRT plans that will be delivered on patients still undergo PSQA. The inclusion of the predictive GPR model helps mitigate the occurrence of failing beams through early detection and preemptive replanning, increasing the likelihood of the plan to pass, reducing repetitive machine measurements, and helping to facilitate same‐day HyperArc delivery through efficient measurement‐based PSQA.

Li et al.^11^ were among the first to perform predictive GPR for VMAT plans, generating models from a combination of gynecological cancer and head and neck cancer VMAT plans, unlike the model developed in this research which is specialized towards a single treatment site. Furthermore, their work directly predicted the GPR value with statistical regression then with a separate random forest model predicted the outcome as pass or fail. Our work explores a different avenue, choosing not to predict the GPR value or a binary classification, and instead introducing a three‐class problem. Thongsawad et al.^12^ conducted a thorough study for predictive GPR using 106 head and neck VMAT plans where their models required non‐negligible feature selection and preprocessing, removing the inherent sequential nature of the VMAT arc, a component that this work valued heavily. The use of a LSTM in this research the complete sequential data of the VMAT arc is considered by the model. Additionally, their work was a feasibility study where they did not implement the model into their clinic. Noblet et al.^13^ did demonstrate a fully TPS integrated machine learning tool to predict GPR for VMAT as well as providing guided re‐optimization suggestions to improve passing likelihood. Their work binarized the output of the model into pass or fail, as opposed to our model which provides a three‐class solution which more accurately represents a realistic clinical workflow. Furthermore, Noblet et al.’s selected model achieved an AUROC of 0.88 with a sensitivity and specificity exceeding 50% and 90%, respectively. We achieved slightly higher AUROC values of 0.89, 0.91, and 0.98 as well as sensitivity and specificity values exceeding 70% and 80%, respectively, an improved sensitivity but slightly worse specificity.

This research is not without limitations, with the most prominent being that the data used was relatively small, from a single institution, and imbalanced. The number of samples is understandable considering the specificity of the model to be used only for HyperArc plans, furthermore as the predictive GPR model is used clinically and more HyperArc SRS/SRT plans are generated and treated, these plans will be used to further refine the model. The imbalanced data, albeit common in research involving GPR, was circumvented by under‐sampling the majority class and over‐sampling the minority class with SMOTE. SMOTE poses a unique limitation where the synthetic data generated is mathematically similar to the real‐world data but has the potential to generate control point data that is not machine deliverable. The SMOTE algorithm maintains the extremes of the data, but it has no sense of the physical limitations of the LINAC. More directly, each synthetic control point is physically possible for the LINAC, but the sequence of those control points might not be possible. Furthermore, the implementation of SMOTE prior to data splitting increases the risk of data leakage between the training and testing sets. Due to the limited data, this step was necessary in order to have a sufficient number of samples within the minority class for the algorithm to work as intended. Increasing the amount of real‐world data within the minority classes would greatly improve the robustness of this model.

Ongoing work is being done to gather additional clinical data to train similar models at different treatment sites, including rapid delivery of lung stereotactic body radiotherapy.^21^, ^22^ Furthermore, the developed ESAPI software and Python model training code is being further refined to eventually be released as free and open‐source software to allow easier dissemination among the medical physics community and to encourage other institutions and clinics to develop their own models. The clinical performance of the model is actively being monitored. Currently, there is insufficient data to draw any substantial conclusions.

Online adaptive radiation therapy is booming in popularity and exposure with modalities such as Magnetic Resonance‐guided LINACs^23^ and Varian's Ethos delivery system.^24^ The topic of performing measurement based PSQA for these same‐day adapted plans is frequently debated. Although the work presented here does not provide a complete solution to this dilemma, the processes and methodologies provide a starting point towards the types of solutions that might be developed in the near future for predictive PSQA models for online adaptive deliveries when the patient is on the table. As these techniques continue to develop, it is the job of the medical physicist to continuously assess the validity and safety of the proposed improvements including phantom less PSQA tools as presented here.

## 5 CONCLUSION

Machine learning continues to establish itself as a fundamental tool in the ever‐changing landscape that is clinical medical physics. Herein a fully TPS integrated machine learning model is developed and deployed to predict the gamma passing rates for stereotactic HyperArc treatments of single and multiple brain metastases. This tool greatly supplements the current PSQA workflow by providing planners and physicists with a quick assessment of the likelihood of a stereotactic plan to pass PSQA criteria a priori. Due to the straightforward data processing and chosen machine learning model, the processes taken throughout this research are easily reproducible and can be expanded to other treatment sites. Other clinics and institutions are encouraged to follow similar steps for simple, easy to implement, predictive GPR models to improve their PSQA workflows for the management of their complex and difficult cancer patients in a timely manner.

## AUTHOR CONTRIBUTIONS

Damodar Pokhrel and Shane McCarthy conceived this research project. Shane McCarthy developed the machine learning model and collected and analyzed the data. Brent Harrison provided machine learning and data mining expertise. Damodar Pokhrel generated all clinical HyperArc brain SRS/SRT plans and provided clinical expertise and supervision of the research. Shane McCarthy drafted preliminary manuscript and all co‐authors revised and approved the final manuscript for submission.

## CONFLICT OF INTEREST STATEMENT

The authors declare no conflicts of interest.

## Data Availability

No data available due to privacy/ethical restrictions.
